# Intensive care unit versus high-dependency care unit for mechanically ventilated patients with pneumonia: a nationwide comparative effectiveness study

**DOI:** 10.1016/j.lanwpc.2021.100185

**Published:** 2021-07-05

**Authors:** Hiroyuki Ohbe, Yusuke Sasabuchi, Hayato Yamana, Hiroki Matsui, Hideo Yasunaga

**Affiliations:** 1 Department of Clinical Epidemiology and Health Economics, School of Public Health, The University of Tokyo, 7-3-1 Hongo, Bunkyo-ku, Tokyo, 1130033, Japan; 2 Data Science Centre, Jichi Medical University, 3311-1 Yakushiji, Shimotsuke, Tochigi Prefecture, 3290498, Japan

**Keywords:** high-dependency care unit, intensive care unit, mechanical ventilation, pneumonia, comparative effectiveness

## Abstract

**Background:**

Many mechanically ventilated patients in Japan are treated in high-dependency care units (HDUs) rather than intensive care units (ICUs). HDUs can provide intermediate-level care with reduced costs; however, there is limited evidence on whether mechanically ventilated patients should be treated in the ICU or HDU.

**Methods:**

This was a comparative effectiveness study using a nationwide administrative database in Japan. We identified mechanically ventilated patients with pneumonia in ICU or HDU on the day of admission in the Japanese Diagnosis Procedure Combination inpatient database from April 2014 to March 2019. The primary outcome was 30-day in-hospital mortality. Propensity score matching analysis was performed to compare this outcome between patients treated in the ICU and HDU. The robustness of the analyses was evaluated with multivariable regression, overlap weighting, and instrumental variable analyses.

**Findings:**

Of 14,859 mechanically ventilated patients with pneumonia, 7,528 (51%) were treated in the ICU and 7,331 (49%) were treated in the HDU. After propensity score matching, patients treated in the ICU had significantly lower 30-day in-hospital mortality than did those treated in the HDU (24.0% vs. 31.2%; difference, −7.2%; 95% confidence interval, −10.0% to −4.4%). The multivariable regression, overlap weighting, and instrumental variable analyses showed a similar direction and magnitude of association.

**Interpretation:**

Critical care for mechanically ventilated patients with pneumonia in the ICU was associated with a 7.2% decrease in 30-day in-hospital mortality vs. care in the HDU. Residual confounding may still play a role in the effect estimates.

**Funding:**

This study received funding from Ministry of Health, Labour and Welfare, Japan, and Ministry of Education, Culture, Sports, Science and Technology, Japan.

**Research in context**

Evidence before this study

Before conducting the present study, we hypothesized that critical care for mechanically ventilated patients with pneumonia in the HDU may be associated with increased mortality than care in the ICU. As such, our initial search was aimed at identifying comparative effectiveness studies that compared the level of critical care among mechanically ventilated patients with pneumonia. We searched Medline via PubMed with the terms (“High Dependency Care Unit” or “Intermediate Care Unit” or “Step Down Unit”) and (“Intensive Care Unit”) and (“Pneumonia”) and (“Mechanical Ventilation”) between 1 January 1990 and 31 December 2020, without any language restriction. Of the 117 search results, no study had compared the mortality between patients treated in the ICU and HDU. Thus, whether patients with mechanical ventilation should be treated in the ICU or the HDU remains unclear.

Added value of this study

The current study is, to the best of our knowledge, the first real-world clinical study that has compared mortality between mechanically ventilated patients with pneumonia treated in the ICU and HDU. As our study is not a randomized controlled trial, several potential biases exist. However, we find that critical care for mechanically ventilated patients with pneumonia in ICU was associated with a 7.2% decrease in 30-day in-hospital mortality compared with care in HDU.

Implications of all the available evidence

This study provided evidence that mechanically ventilated patients be treated in the ICU rather than in the HDU or general ward. Establishing regulations for HDUs to be in the same hospitals as ICUs may increase opportunities for mechanically ventilated patients to be treated in the ICU and facilitate the appropriate allocation of critical care resources.

Alt-text: Unlabelled box

## Introduction

Intensive care units (ICUs) are a limited and expensive resource. Appropriate utilization of ICU beds is essential but complex and difficult to achieve.^1^ High-dependency care units (HDUs), also called “intermediate care units” or “step-down units,” are areas where patient care levels and costs are between the levels found in the ICU and in the general ward.^1^^,^ ^2^^,^ ^3^ HDUs may be used in different ways in the continuum of inpatient critical care, and, worldwide, HDUs are increasing widely to optimize critical care resource allocation.^2^^,^ ^3^^,^ ^4^^,^ ^5^^,^ ^6^

In general, patients requiring mechanical ventilation or life-sustaining interventions are treated in the ICU rather than the HDU.^1^ However, a recent study in Japan showed that a considerable number of mechanically ventilated patients were treated in the HDU under normal conditions.^7^ In Japan, the number of ICU beds per capita is the lowest among developed countries (about five beds per 100,000 population), whereas the total number of hospital beds is the highest, and the number of the HDU beds is about eight beds per 100,000 population.^8^^,^ ^9^ The low prevalence of ICU services and the relatively high prevalence of HDU services in Japan may stem from historical tradition, insufficient public and social awareness, scarce government funding, and an underdeveloped educational system.^10^ In addition to Japan, some other locations, such as Hong Kong and Israel, also treat many patients with mechanical ventilation outside the ICU.^11^^,^ ^12^^,^ ^13^^,^ ^14^ Mechanically ventilated patients treated outside the ICU have a poor prognosis.^7^^,^ ^11^^,^ ^12^^,^ ^13^^,^ ^14^

In addition to the situation under normal conditions, mechanical ventilation in the HDU is a possible alternative to mechanical ventilation in the ICU in response to surges in demand for care for critically ill patients during a disaster or pandemic.^15^^,^ ^16^ During the massive coronavirus disease 2019 (COVID-19) pandemic, the number of critically ill patients with COVID-19 exceeded pre-pandemic ICU bed capacity in several areas, such as New York in the United States and Lombardy in Italy.^17^^,^ ^18^ Although the healthcare systems in these areas increased ICU capacity for critical care utilization, including by providing critical care outside the ICU, the mortality of critically ill patients with COVID-19 was high.^17^^,^ ^18^ Therefore, it is clinically relevant to verify whether patients with mechanical ventilation can be treated in the HDU rather than the ICU.

However, evidence to answer this clinical question is scarce. Recent ICU admission guidelines suggest that patients with invasive mechanical ventilation be treated in the ICU, although this recommendation is weak because of a low certainty of evidence.^1^ Previous epidemiological studies have shown that mechanically ventilated patients treated in the HDU or general ward had increased mortality compared with those treated in the ICU.^7^^,^ ^11^^,^ ^12^^,^ ^13^^,^ ^14^ However, these studies were not comparative effectiveness studies and had considerable limitations in terms of comparing the outcomes between the two groups, mainly because of residual confounding. Further studies are thus warranted to examine whether patients with mechanical ventilation should be treated in the ICU or the HDU.

Therefore, to assess the outcomes of mechanically ventilated patients with pneumonia treated in the HDU vs. those treated in the ICU, we conducted a comparative effectiveness study using recommended methods to account for confounding by indication using a nationwide inpatient database.

## Methods

### Data source

This comparative effectiveness study was conducted using a nationwide administrative database in Japan. The Institutional Review Board of The University of Tokyo approved this study (approval number, 3501-3; December 25, 2017). The procedures used in this study adhere to the tenets of the Declaration of Helsinki.

We used the Japanese Diagnosis Procedure Combination inpatient database, which contains discharge abstracts and administrative claims data from more than 1,200 acute-care hospitals in Japan that voluntarily contribute to the database.^19^ This database includes the following patient-level data for all hospitalizations: age; sex; diagnoses recoded with International Classification of Diseases, Tenth Revision (ICD-10) codes; daily procedures recorded using Japanese medical procedure codes; daily drug administrations; and admission and discharge status. A previous validation study for this database showed high specificity and moderate sensitivity for diagnoses and high specificity and sensitivity for procedures.^20^

We also used facility information and statistics data from the Survey of Medical Institutions 2017.^21^ We combined this information with the Japanese Diagnosis Procedure Combination inpatient database data using a specific hospital identifier. The Survey of Medical Institutions included the hospital ZIP code, type of ward (e.g., general, ICU, or HDU), number of hospital beds in each ward, and hospital type (i.e., teaching hospital, tertiary emergency hospital, or academic hospital).

### Study population

Using the Japanese Diagnosis Procedure Combination inpatient database from April 2014 to March 2019, we identified patients hospitalized for pneumonia who were admitted to the ICU or HDU on the day of hospital admission and who received invasive mechanical ventilation on the day of admission. We defined patients hospitalized for pneumonia as those with a diagnosis of pneumonia (ICD-10 codes: A48.1, B01.2, B05.2, B37.1, B59, J10.0, J11.0, or J12–J18) as the admission-precipitating diagnosis listed in the discharge abstract. We limited the study population to patients with pneumonia because a previous study found that the main population of mechanically ventilated patients in the HDU were those with pneumonia.^7^ The definition of an ICU in this study was a separate unit providing critical care services with at least one physician on site 24 hours per day, at least two intensivists working full-time, around-the-clock nursing, the equipment necessary to care for critically ill patients, and a nurse-to-patient ratio of 1 to 2. The definition of an HDU in this study was almost same as an ICU, except HDUs were not required to employ intensivists and the requirement for the nurse-to-patient ratio was reduced to 1 to 4 or 1 to 5. We present the Japanese procedure codes used to define ICUs and HDUs in Supplemental Table 1. We excluded patients aged < 15 years, those in hospitals for which Survey of Medical Institutions 2017 data could not be combined, and patients with missing data on the severity of pneumonia, which attending physicians were required to enter for all patients admitted for pneumonia.

Patients who were admitted to the ICU on the day of hospital admission were defined as the ICU group. Patients who were admitted to the HDU on the day of hospital admission were defined as the HDU group.

### Outcomes and covariates

The primary outcome was 30-day in-hospital mortality. The secondary outcomes were ICU/HDU mortality, length of hospital stay, length of ICU/HDU stay, ICU/HDU-free days, length of mechanical ventilation, ventilator-free days, total hospitalization costs (with 1 US dollar equivalent to 110 Japanese yen), acute respiratory distress syndrome after admission, and catheter-related bloodstream infection after admission. The number of ICU/HDU-free days was defined by counting each day after ICU/HDU admission until day 30 that patients were alive and free of ICU/HDU stay; this variable was entered as zero for patients who died by day 30. The number of ventilator-free days was defined by counting each day after initial mechanical ventilation until day 30 that patients were alive and free of mechanical ventilation; this variable was entered as zero for patients who died by day 30.^22^ Acute respiratory distress syndrome was defined as ICD-10 code J80 appearing as a complication after admission. Catheter-related bloodstream infection was defined as ICD-10 code T814 or T827 with “catheter-related bloodstream infection,” “catheter infection,” or “central venous catheter infection” in Japanese text appearing as a complication after admission.

The covariates were age, sex, smoking history, body mass index at admission, Charlson comorbidity index score, comorbidity of congestive heart failure, malignancy, and metastatic solid tumor, physical function at admission measured by the Barthel Index score, cognitive function before admission, home medical care before admission, ambulance use, admission on a weekend (i.e., on Saturday or Sunday), location before hospitalization, cause of pneumonia, community-acquired or hospital-acquired pneumonia, severity of pneumonia at admission, organ support therapies on the day of admission, and hospital characteristics.

Cause of pneumonia was categorized as typical bacteria (ICD-10 codes: J13, J14, J15.0–J15.6, J15.8, J15.9, or J17.0); atypical bacteria (A48.1, J15.7, or J16.0); virus (B01.2, B05.2, J10.0, J11.0, J12, or J17.1); fungi, parasite, or other (B37.1, B59, J16.8, J17.2, J17.3, or J17.8); or pathogen not confirmed (J18). Severity of pneumonia at admission was recorded using the A-DROP (age, dehydration, respiration, disorientation, and blood pressure) system, a modified simple version of the CURB-65 (confusion, uremia, respiratory rate, blood pressure, age ≥ 65 years) score.^23^ The A-DROP system includes the following variables: blood urea nitrogen ≥ 21 mg/dl or dehydration, oxygenation (SpO2 > 90% in room air, SpO2 > 90% with FiO2 < 35%, or SpO2 > 90% with FiO2 ≥ 35%), level of consciousness, and systolic blood pressure. Following the severity rating system from the Japanese Respiratory Society, immunocompromised status and C-reactive protein ≥ 20 mg/dl or unilateral lung infiltration more than two-thirds on chest radiography were also recorded as indicators of the severity of pneumonia.^24^

### Statistical analysis

Our primary approach to compare the outcomes between the ICU and HDU groups was a propensity score matching analysis.^25^ A multivariable logistic regression model using all the covariates listed in Table 1 was employed to compute the propensity scores for patients who were admitted to the ICU on the day of admission. One-to-one nearest-neighbor matching without replacement was then performed for the estimated propensity scores using a caliper width set at 20% of the standard deviation of the propensity scores.^25^ To assess the performance of the matching, the covariates were compared using standardized mean differences, with absolute standardized mean differences ≤ 10% considered to denote negligible imbalances between the two groups.^26^

**Table 1:** Baseline characteristics before and after propensity score matching

	Before propensity score matching			After propensity score matching		
	ICU	HDU		ICU	HDU	
Characteristics	(n = 7,528)	(n = 7,331)	ASD	(n = 4,982)	(n = 4,982)	ASD
Age, years, mean (SD)	74 (15)	77 (13)	23	75 (14)	75 (14)	1
Male, n (%)	5,081 (67)	4,688 (64)	8	3,270 (66)	3,298 (66)	1
Smoking history, n (%)						
Nonsmoker	3,621 (48)	3,719 (51)	5	2,483 (50)	2,463 (49)	1
Current/past smoker	2,404 (32)	2,352 (32)	0	1,619 (32)	1,613 (32)	0
Unknown	1,503 (20)	1,260 (17)	7	880 (18)	906 (18)	1
Body mass index at admission, kg/m^2^, n (%)						
< 18.5	1,975 (26)	2,085 (28)	5	1,356 (27)	1,335 (27)	1
18.5–24.9	3,385 (45)	3,034 (41)	7	2,130 (43)	2,166 (43)	2
25.0–29.9	861 (11)	792 (11)	2	564 (11)	559 (11)	0
≥ 30.0	332 (4)	278 (4)	3	214 (4)	217 (4)	0
Missing	975 (13)	1,142 (16)	8	718 (14)	705 (14)	1
Charlson comorbidity index, mean (SD)	1.4 (1.4)	1.4 (1.4)	1	1.4 (1.4)	1.4 (1.4)	2
Comorbidities, n (%)						
Congestive heart failure	2,330 (31)	2,463 (34)	6	1,663 (33)	1,661 (33)	0
Malignancy	520 (7)	531 (7)	1	339 (7)	344 (7)	0
Metastatic solid tumor	67 (1)	51 (1)	2	31 (1)	40 (1)	2
Physical function at admission, n (%)						
Total/severe dependence (Barthel Index 0–60)	5,449 (72)	5,514 (75)	6	3,697 (74)	3,712 (75)	1
Slight/moderate dependence (Barthel Index 61–99)	122 (2)	174 (2)	5	92 (2)	87 (2)	1
Independent (Barthel Index 100)	573 (8)	565 (8)	0	362 (7)	357 (7)	0
Missing	1,384 (18)	1,078 (15)	10	831 (17)	826 (17)	0
Cognitive function before admission, n (%)						
No dementia	5,472 (73)	4,939 (67)	12	3,485 (70)	3,517 (71)	1
Mild dementia	1,120 (15)	1,290 (18)	7	811 (16)	768 (15)	2
Moderate/severe dementia	936 (12)	1,102 (15)	8	686 (14)	697 (14)	1
Home medical care before admission, n (%)	733 (10)	949 (13)	10	559 (11)	549 (11)	1
Ambulance use, n (%)	6,250 (83)	5,856 (80)	8	4,034 (81)	4,056 (81)	1
Admission on a weekend, n (%)	1,992 (26)	1,902 (26)	1	1,321 (27)	1,334 (27)	1
Location before hospitalization, n (%)						
Home	5,872 (78)	5,706 (78)	0	3,906 (78)	3,924 (79)	1
Other hospitals	1,084 (14)	754 (10)	13	607 (12)	610 (12)	0
Nursing home	572 (8)	871 (12)	15	469 (9)	448 (9)	1
Cause of pneumonia, n (%)						
Typical bacteria	3,770 (50)	3,542 (48)	4	2,506 (50)	2,527 (51)	1
Atypical bacteria	128 (2)	77 (1)	6	57 (1)	65 (1)	1
Viruses	127 (2)	94 (1)	3	73 (1)	71 (1)	0
Fungus, parasite, and others	142 (2)	105 (1)	4	82 (2)	93 (2)	2
Pathogen-nonconfirmed	3,361 (45)	3,513 (48)	7	2,264 (45)	2,226 (45)	2
Community-acquired pneumonia, n (%)	6,735 (89)	6,663 (91)	1	4,481 (90)	4,514 (91)	2
Severity of pneumonia, n (%)						
Blood urea nitrogen >21 mg/dl or dehydration	5,137 (68)	4,634 (63)	11	3,246 (65)	3,291 (66)	2
Oxygenation						
SpO_2_ > 90% in room air	1,133 (15)	992 (14)	4	745 (15)	743 (15)	0
SpO_2_ > 90% in FiO_2_ < 35%	1,541 (20)	1,978 (27)	15	1,189 (24)	1,137 (23)	3
SpO_2_ > 90% in FiO_2_ ≥ 35%	4,854 (64)	4,361 (59)	10	3,048 (61)	3,102 (62)	2
Impaired consciousness	4,402 (58)	3,897 (53)	11	2,732 (55)	2,753 (55)	1
Systolic blood pressure < 90 mmHg	2,328 (31)	1,833 (25)	13	1,276 (26)	1,332 (27)	3
A-DROP score, mean (SD)	1,417 (19)	1,160 (16)	8	812 (16)	831 (17)	1
Immunocompromised, n (%)	3,819 (51)	3,086 (42)	17	2,245 (45)	2,348 (47)	4
CRP ≥ 20mg/dl or lung infiltration, n (%)	3.1 (1.3)	3.0 (1.2)	5	3.0 (1.2)	3.0 (1.3)	2
Organ support therapies on the day of admission, n (%)						
Dopamine	930 (12)	600 (8)	14	504 (10)	514 (10)	1
Dobutamine	434 (6)	185 (3)	16	160 (3)	171 (3)	1
Noradrenaline	2,463 (33)	1,007 (14)	46	919 (18)	991 (20)	4
Adrenaline	785 (10)	699 (10)	3	454 (9)	464 (9)	1
Vasopressin	323 (4)	86 (1)	19	71 (1)	84 (2)	2
Cardiac massage	550 (7)	650 (9)	6	388 (8)	382 (8)	0
Defibrillation	110 (1)	72 (1)	4	57 (1)	58 (1)	0
Red blood cell transfusion	369 (5)	161 (2)	15	124 (2)	146 (3)	2
Fresh frozen plasma transfusion	142 (2)	35 (0)	13	30 (1)	35 (1)	1
Platelet transfusion	59 (1)	18 (0)	8	14 (0)	15 (0)	0
Albumin	509 (7)	159 (2)	22	131 (3)	151 (3)	2
Renal replacement therapy	357 (5)	153 (2)	15	132 (3)	134 (3)	0
Extracorporeal membrane oxygenation	48 (1)	7 (0)	9	4 (0)	7 (0)	1
Hospital characteristics, n (%)						
Teaching hospital	7,059 (94)	6,742 (92)	7	4,618 (93)	4,613 (93)	0
Tertiary emergency hospital	4,381 (58)	4,898 (67)	18	2,973 (60)	2,920 (59)	2
Academic hospital	1,595 (21)	564 (8)	39	512 (10)	562 (11)	3
Total number of hospital beds						
Low (24–381 beds)	1,750 (23)	2,025 (28)	10	1,430 (29)	1,438 (29)	0
Medium low (382–522 beds)	1,703 (23)	1,966 (27)	10	1,279 (26)	1,248 (25)	1
Medium high (523–680 beds)	1,868 (25)	1,836 (25)	1	1,222 (25)	1,208 (24)	1
High (681–1,334 beds)	2,207 (29)	1,504 (21)	21	1,051 (21)	1,088 (22)	2

ICU, intensive care unit; HDU, high-dependency care unit; ASD, absolute standardized mean difference; SD, standard deviation; A-DROP, age, dehydration, respiration, disorientation, and blood pressure; CRP, C-reactive protein

After the propensity score matching, the outcomes for the two groups were assessed through a generalized linear model. Differences and their 95% confidence intervals were calculated with generalized linear models using the identity link function, irrespective of outcome types. For 30-day in-hospital mortality, we also generated Kaplan–Meier curves and performed log-rank tests in the matched cohort.

### Sensitivity analyses

We performed three sensitivity analyses to confirm the robustness of our primary outcome analysis by applying different statistical models. First, we performed a traditional multivariable regression analysis using the primary outcome as the dependent variable and ICU admission and all the covariates as the independent variables through a generalized linear model.

Second, we performed an overlap weighting analysis.^27^ Overlap weighting analysis emphasizes the target population with the most overlap in observed characteristics between two groups. We calculated the standardized mean differences to assess the balance of covariates between the two groups. We used a weighted generalized linear model to compare the primary outcome.

Third, we performed an instrumental variable analysis to address unmeasured confounders. We chose differential distance as the instrumental variable.^28^ Differential distance was calculated as the driving distance from a patient's residence to the nearest hospital with relatively low ICU use (< 50% of patients treated in the ICU) minus the driving distance from the patient's residence to the nearest hospital with relatively high ICU use (≥ 50% of patients treated in the ICU). We included the instrumental variable of differential distance as a continuous variable, with truncation at the first and 99th percentiles. To assess the validity of differential distance as an instrumental variable, we confirmed that continuous instrumental variable was highly correlated with ICU admission on the day of hospital admission (*F* statistic > 10). To examine whether the covariates were associated with differential distance, we calculated the absolute standardized mean differences between patients with a differential distance of ≥ 0 km (living nearer to a hospital relatively high ICU use) or of < 0 km (living nearer to a hospital with relatively low ICU use). We used a two-stage residual inclusion estimation framework for the instrumental variable analysis.^29^

### Subgroup analyses

We performed nine subgroup analyses for primary outcome. First, the characteristics between hospitals with only ICU beds and hospitals with only HDU beds may be different. Therefore, we performed an analysis excluding patients who were admitted to hospitals with only ICU beds or only HDU beds. Second, hospitals with both ICU beds and HDU beds may use the HDU as a step-down unit for ICU, so the choice of the ICU or the HDU may itself be a major confounder in such hospitals. Therefore, we performed an analysis excluding patients admitted to hospitals with both ICU beds and HDU beds. Third, patients with a significantly lower probability of recovery who die on the day of hospital admission may have a lower priority for ICU admission.^1^ Therefore, we performed an analysis excluding patients who died on the day of hospital admission. ICUs with ICU management fees 1 or 2 have more critical care resources than do ICUs with emergency and critical care unit management fees 2 or 4 or ICU management fees 3 or 4. Therefore, for the fourth and fifth subgroups, we separately compared outcomes between patients who were admitted to ICUs with ICU management fees 1 or 2 vs. HDUs and between patients who were admitted to ICUs either with emergency and critical care unit management fees 2 or 4 or with ICU management fees 3 or 4 vs. HDUs. HDUs with emergency and critical care unit management fees 1 or 3 have more critical care resources than do HDUs with HDU management fees 1 or 2. Therefore, for the sixth and seventh subgroups, we separately compared the outcomes between patients who were admitted to ICUs vs. HDUs with emergency and critical care unit management fees 1 or 3 and between patients who were admitted to ICUs vs. HDUs with HDU management fees 1 or 2. For the eighth and ninth subgroups, we separately analysed patients with pathogen-confirmed pneumonia (typical bacteria, atypical bacteria, virus, fungi, parasite, or other) and those with pathogen-nonconfirmed pneumonia.

All analyses were performed using Stata/MP 16.0 software (StataCorp). Continuous variables were presented as means and standard deviations (SDs), and categorical variables were described using numbers and percentages. All the generalized linear models in the outcome analyses were accompanied by cluster-robust standard errors with hospitals as the clusters. All reported *P* values were two-sided, and *P* < .05 was considered statistically significant.

### Role of the funding source

This work was supported by grants from the Ministry of Health, Labour and Welfare, Japan (19AA2007 and H30-Policy-Designated-004) and the Ministry of Education, Culture, Sports, Science and Technology, Japan (17H04141). The funding sources had no involvement in the present study.

## Results

**Fig. 1:**
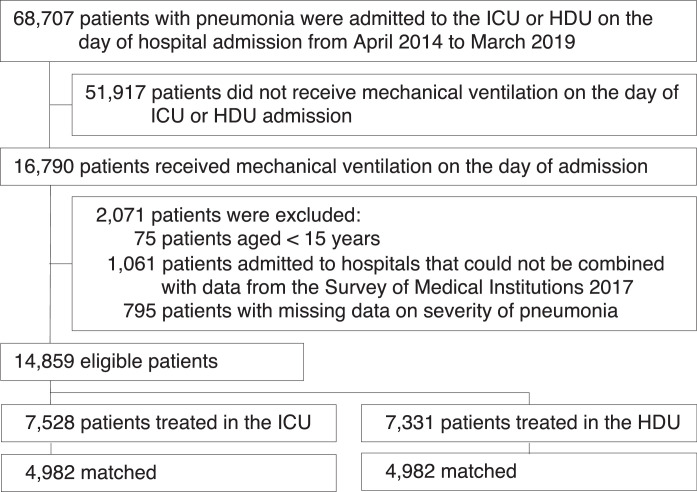
Flowchart of patient selection ICU, intensive care unit; HDU, high-dependency care unit

A total of 14,859 mechanically ventilated patients with pneumonia who were admitted to 689 hospitals with ICU and/or HDU beds were enrolled during the five years study period. Of these patients, 7,528 (51%) were treated in the ICU and 7,331 (49%) were treated in the HDU on the day of admission (Figure 1). Of the 14,859 patients in the 689 hospitals with ICU and/or HDU beds, 1,617 patients were in 159 hospitals with only ICU beds, and 1,683 patients were in 169 hospitals with only HDU beds; in the 361 hospitals with both ICU beds and HDU beds, 5,911 patients were treated in the ICU, and 5,648 were treated in the HDU. Of the 7,528 patients admitted to the ICU on the day of admission, 1,689/7,528 (22%) were subsequently transferred to the HDU. Of the 7,331 patients who were admitted to the HDU on the day of admission, 244/7,331 (3%) were subsequently transferred to the ICU.

Table 1 shows the baseline characteristics before and after propensity score matching. In the original cohort, patients in the ICU group tended to be younger, have been transferred from another hospital, have higher severity of pneumonia, require organ support therapies, be admitted to an academic hospital, and be admitted to a hospital with high total number of hospital beds, whereas patients in the HDU group tended to have dementia, have been transferred from a nursing home, and be admitted to a tertiary emergency hospital. The numbers of patients with each pneumonia code are presented in Supplemental Table 2. The discrimination ability, as measured by the area under the receiver operating characteristic curve for estimated propensity scores, was 0.722. The distributions of propensity scores before and after matching are shown in Supplemental Figures 1 and 2. One-to-one propensity score matching created 4,982 matched pairs. After propensity score matching, the patients’ characteristics were well balanced between the two groups (Table 1 and Supplemental Figure 3).

**Fig. 2:**
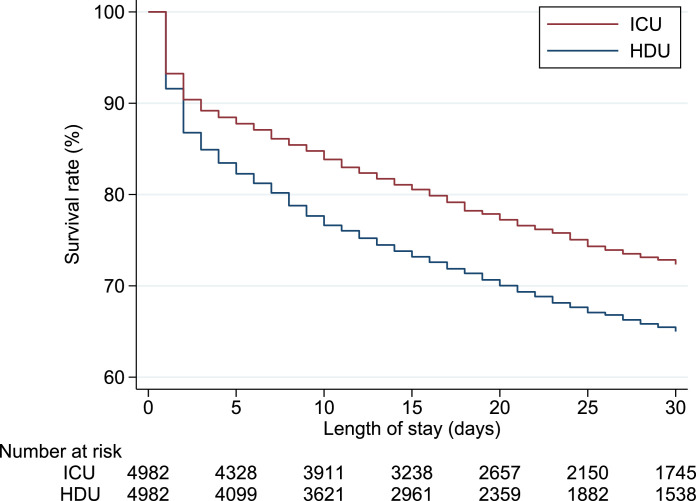
Kaplan–Meier survival plots for mechanically ventilated patients with pneumonia treated in the ICU vs. HDU in propensity score-matched cohorts There was a statistically significant difference in 30-day in-hospital mortality between the two groups (log-rank test, P value < .001). HDU, high-dependency care unit; ICU, intensive care unit

Table 2 shows the outcomes before and after propensity score matching. After propensity score matching, patients treated in the ICU had significantly lower 30-day in-hospital mortality than did those treated in the HDU (24.0% vs. 31.2%; difference, −7.2%; 95% confidence interval, −10.0% to −4.4%). Kaplan–Meier analysis with the log-rank test showed a statistically significant difference in 30-day in-hospital mortality between the two groups (*P* value < .001) (Figure 2). Compared with patients treated in the HDU, those in the ICU group had significantly lower ICU/HDU mortality, longer lengths of hospital stay, longer ICU/HDU-free days, longer mechanical ventilation-free days, and higher hospitalization costs.

**Table 2:** Outcomes before and after propensity score matching

	Before propensity score matching		After propensity score matching			
	ICU	HDU	ICU	HDU	Difference	
Outcomes	(n=7,528)	(n=7,331)	(n = 4,982)	(n = 4,982)	(95% CI)	*P* value
30-day in-hospital mortality	1,867 (24.8)	2,344 (32.0)	1,196 (24.0)	1,555 (31.2)	-7.2 (-10.0 to -4.4)	< .001
ICU/HDU mortality	1,165 (15.5)	1,575 (21.5)	741 (14.9)	1,061 (21.3)	-6.4 (-9.3 to -3.6)	< .001
Length of hospital stay, days	31 (40)	26 (33)	30 (34)	27 (34)	3.2 (1.4 to 5.1)	.001
ICU/HDU-free days, days	17 (11)	15 (12)	18 (11)	15 (11)	2.3 (1.6 to 3.1)	< .001
MV-free days, days	15 (12)	14 (12)	15 (12)	14 (12)	1.4 (0.7 to 2.1)	< .001
Total hospitalization cost, USD	19,240 (17,563)	13,842 (13,541)	17,830 (16,028)	14,653 (14,319)	3,176 (2,191 to 4,162)	< .001
ARDS after admission	106 (1.4)	59 (0.8)	54 (1.1)	48 (1.0)	0.1 (-0.3 to 0.5)	.58
CRBSI after admission	16 (0.2)	11 (0.2)	12 (0.2)	10 (0.2)	0.0 (-0.2 to 0.2)	.70

Continuous variables are presented as means and standard deviations, and categorical variables are described using numbers and percentages.

ICU, intensive care unit; HDU, high-dependency care unit; CI, confidence interval; MV, mechanical ventilation; USD, United States dollars; ARDS, acute respiratory distress syndrome; CRBSI, catheter related blood stream infection

The results of the three sensitivity analyses for 30-day in-hospital mortality showed a similar direction and magnitude to those in the primary analysis (Table 3). In the overlap weighting analysis, the patients’ characteristics were perfectly balanced between the two groups (Supplemental Figure 4). In the instrumental variable analysis, 4,882/7,103 (69%) patients who lived nearer to a hospital with relatively high ICU use were admitted to the ICU on the day of admission and 2,646/7,756 (34%) patients lived nearer to a hospital with relatively low ICU use were admitted to the ICU on the day of admission. The continuous instrumental variable of differential distance was strongly associated with ICU admission on the day of admission (*F* statistic = 707). The characteristics were well balanced between patients with a differential distance of ≥ 0 km and those with a differential distance of < 0 km, except for hospital characteristics (Supplemental Table 3).

**Table 3:** Results of sensitivity and subgroup analyses for 30-day in-hospital mortality

	Number of patients		Difference	
Analyses	ICU	HDU	(95% CI)	*P* value
Overall cohort				
Propensity score matching	4,982	4,982	-7.2 (-10.0 to -4.4)	< .001
Multivariable regression	7,528	7,331	-7.1 (-8.7 to -5.5)	< .001
Overlap weight	7,528	7,331	-7.1 (-9.9 to -4.4)	< .001
Instrumental variable	7,528	7,331	-10.1 (-14.3 to -5.8)	< .001
Subgroup 1: Excluding patients who were admitted to hospitals with only ICU beds or only HDU beds				
Propensity score matching	4,180	4,180	-6.3 (-9.5 to -3.1)	< .001
Multivariable regression	5,911	5,648	-7.2 (-9.1 to -5.4)	< .001
Overlap weight	5,911	5,648	-7.3 (-10.5 to -4.1)	< .001
Instrumental variable	5,911	5,648	-14.1 (-20.3 to -7.8)	< .001
Subgroup 2: Excluding patients who were admitted to hospitals with both ICU beds and HDU beds				
Propensity score matching	913	913	-5.3 (-11.0 to 0.5)	.075
Multivariable regression	1,617	1,683	-4.7 (-8.0 to -1.5)	.005
Overlap weight	1,617	1,683	-4.8 (-11.1 to 1.5)	.13
Instrumental variable	1,617	1,683	-3.7 (-8.5 to 1.1)	.14
Subgroup 3: Excluding patients who died on the day of admission				
Propensity score matching	4,935	4,935	-6.5 (-8.4 to -4.7)	< .001
Multivariable regression	7,061	6,602	-6.3 (-7.8 to -4.7)	< .001
Overlap weight	7,061	6,602	-6.3 (-8.2 to -4.5)	< .001
Instrumental variable	7,061	6,602	-9.3 (-14.0 to -4.6)	< .001
Subgroup 4: ICUs with ICU management fees 1 or 2 vs. HDUs				
Propensity score matching	1,026	1,026	-8.7 (-12.9 to -4.5)	< .001
Multivariable regression	1,105	7,331	-8.6 (-11.4 to -5.9)	< .001
Overlap weight	1,105	7,331	-8.9 (-12.3 to -5.5)	< .001
Instrumental variable	1,105	7,331	-12.6 (-22.8 to -2.5)	.014
Subgroup 5: ICUs either with emergency and critical care unit management fees 2 or 4 or with ICU management fees 3 or 4 vs. HDUs				
Propensity score matching	4,546	4,546	-6.6 (-9.5 to -3.8)	< .001
Multivariable regression	6,423	7,331	-6.7 (-8.4 to -5.1)	< .001
Overlap weight	6,423	7,331	-6.8 (-9.6 to -3.9)	< .001
Instrumental variable	6,423	7,331	-9.3 (-13.7 to -4.9)	< .001
Subgroup 6: ICUs vs. HDUs with emergency and critical care unit management fees 1 or 3				
Propensity score matching	2,800	2,800	-7.8 (-12.1 to -3.4)	< .001
Multivariable regression	7,528	4,634	-8.4 (-10.6 to -6.3)	< .001
Overlap weight	7,528	4,634	-8.4 (-12.6 to -4.1)	< .001
Instrumental variable	7,528	4,634	-13.1 (-17.9 to -8.2)	< .001
Subgroup 7: ICUs vs. HDUs with HDU management fees 1 or 2				
Propensity score matching	2,369	2,369	-5.5 (-8.2 to -2.9)	< .001
Multivariable regression	7,528	2,697	-5.2 (-7.3 to -3.2)	< .001
Overlap weight	7,528	2,697	-5.7 (-8.2 to -3.2)	< .001
Instrumental variable	7,528	2,697	-15.6 (-7.8 to 4.7)	.62
Subgroup 8: Pathogen-confirmed pneumonia				
Propensity score matching	2,714	2,714	-6.7 (-9.3 to -4.1)	< .001
Multivariable regression	4,167	3,818	-6.7 (-8.7 to -4.6)	< .001
Overlap weight	4,167	3,818	-6.6 (-9.1 to -4.1)	< .001
Instrumental variable	4,167	3,818	-9.0 (-15.0 to -3.0)	.004
Subgroup 9: Pathogen-nonconfirmed pneumonia				
Propensity score matching	2,223	2,223	-8.4 (-12.7 to -4.1)	< .001
Multivariable regression	3,361	3,513	-7.6 (-9.8 to -5.5)	< .001
Overlap weight	3,361	3,513	-7.7 (-12.1 to -3.4)	0.001
Instrumental variable	3,361	3,513	-12.2 (-18.2 to -6.3)	< .001

ICU, intensive care unit; HDU, high-dependency care unit; CI, confidence interval

The results of the nine subgroup analyses for 30-day in-hospital mortality showed a similar direction and magnitude to those for the full cohort (Table 3).

## Discussion

In this nationwide comparative effectiveness study of 14,859 mechanically ventilated patients with pneumonia, care in the ICU was associated with decreased 30-day in-hospital mortality compared with care in the HDU. This finding persisted after adjusting for potential confounding factors, using instrumental variable analysis to address unmeasured confounders, and conducting several subgroup analyses.

As expected, mechanically ventilated patients treated in the ICU had lower mortality than did those treated in the HDU. These results were consistent with previous epidemiological studies.^7^^,^ ^11^^,^ ^12^^,^ ^13^^,^ ^14^ What is new in this study in contrast to previous studies was that the present study directly compared the ICU vs. the HDU rather than the ICU vs. the general ward and appropriate statistical analyses were used to compare the two groups using nationwide data on a large number of patients.

The difference in mortality between the ICU and HDU groups in our study may be explained by two factors: higher nurse-to-patient ratios and the presence of intensivists in ICUs. The fundamental difference between the ICU and other hospital departments is the capacity to monitor and to react. In our study, Kaplan–Meier curves showed that the mortality difference between patients in the ICU and those in the HDU occurred especially in the first few days after ICU admission, which suggests that the capacity to monitor and to react may have a significant impact on mortality. Critically ill patients, particularly those with mechanical ventilation, require intensive monitoring and create a high workload for nurses.^30^ There is growing evidence that inadequate nurse staffing and increased nurse workload affects the delivery of care and increases the risk of mortality.^31^ Additionally, increasing the ICU nurse-to-patient ratio has certain benefits in terms of mortality.^32^ To date, however, there is a lack of consensus regarding the appropriate nurse-to-patient ratio for the care of patients with mechanical ventilation. Although further studies are warranted, a nurse-to-patient ratio of 1 to 2 would be preferable to one of 1 to 4 for mechanically ventilated patients.

Abundant evidence supports the superiority of a high-intensity intensivist model, in which an intensivist is responsible for day-to-day patient management, in terms of improved mortality.^33^ In critical care facilities without intensivists, the lack of knowledge of the rapidly changing critical care field may delay the implementation of current standards of care and new technologies. A recent study has suggested that the optimal patient-to-intensivist ratio is 1 to 7.5, with significantly increased mortality above and below this ratio.^34^ On the basis of this previous work and our results, it would be preferable to treat mechanically ventilated patients with the involvement of intensivists.

Our results have important clinical implications. First, we provide robust evidence that mechanically ventilated patients should be treated in the ICU rather than in the HDU or general ward. Second, in countries such as the United Kingdom and India, it is mandatory for HDUs to be in the same hospitals as ICUs.^4^^,^ ^6^ Therefore, establishing regulations for HDUs may increase opportunities for mechanically ventilated patients to be treated in the ICU and facilitate the appropriate allocation of critical care resources, resulting improved outcomes. We hope that our results will influence health care providers, hospital administrators, and policy makers to provide better critical care services in the future.

The present study had some limitations. First, we used a multicentre, real-world database in Japan, and there was no standard protocol for critical care admission. Therefore, admission to the HDU rather than the ICU for mechanical ventilation was not random but rather based on the decisions of the attending physician, which may have led to confounding by indication. We attempted to control for measured confounders in the adjusted analyses; however, there still may have been unmeasured confounders. Although we included the severity of pneumonia and organ support therapies as covariates, we were not able to obtain data on well-known ICU severity scores such as the Sequential Organ Failure Assessment score,^35^ the Simplified Acute Physiology Score,^36^ or the Acute Physiology and Chronic Health Evaluation score.^37^ To address unmeasured confounders, we performed an instrumental variable analysis with an appropriate instrumental variable that seemed to satisfy the required assumptions. We also performed an analysis excluding patients admitted to hospitals with both ICU beds and HDU beds. All the analyses showed similar results, suggesting that our findings are robust. Second, according to the concept of progressive patient care,^38^ patients with invasive mechanical ventilation should be treated in the ICU rather than in the HDU. However, about half of the mechanically ventilated patients with pneumonia in our cohort were treated in the HDU, representing the inefficient utilization of ICU beds in Japan. Therefore, the results of this study may not be generalizable to other countries where critical care systems are utilized efficiently, in line with national regulations or ICU admission guidelines.^1^^,^ ^4^^,^ ^6^ Third, length of ICU/HDU stay may have been underestimated because ICU and HDU stays can be billed for only up to 14 days and 21 days, respectively. This difference in duration for reimbursement between the ICU and HDU may have affected the ICU/HDU-free days. Fourth, we were not able to obtain data on withholding or withdrawing life-sustaining therapy or on do-not resuscitate orders, which might affect a patient's prioritization for ICU admission.^1^ However, because all patients in the present study received invasive mechanical ventilation on the day of admission and were admitted to the ICU or HDU on the same day, these patients would not have been at the end-of-life at the time of treatment allocation in emergency and intensive care medicine. Furthermore, because the allocation of patients to the ICU or the HDU occurred on the day of admission in our study, there seems to be no association between treatment allocation and the decision to withdraw life-sustaining therapy, which generally requires several days. Therefore, the lack of data on withholding or withdrawing life-sustaining therapy may not have caused bias in our study.

In conclusion, this study, using a nationwide inpatient database for comparative effectiveness analyses, suggests that care for mechanically ventilated patients with pneumonia in the ICU may be associated with decreased 30-day in-hospital mortality compared with care in the HDU. However, residual confounding may still play a role in the effect estimates in our results. Further studies were warranted to confirm our findings in different critical care systems.

## Ethics approval and consent to participate

The study was approved by the Institutional Review Board of The University of Tokyo (approval number, 3501-3; December 25, 2017). No information allowing the identification of individual patients, hospitals, or physicians was obtained, and the requirement for informed consent was waived because of the anonymous nature of the data.

## Consent for publication

All authors approved the final version submitted for publication.

## Data sharing statement

The datasets analysed in the current study are not publicly available because of contracts with the hospitals providing data to the database.

## Authors’ contributions

HO designed the research; HO and HM conducted the research; HO and HM analysed the data; HO, YS, HY, and HY wrote the paper; and HO had primary responsibility for the final content. All authors read and approved the final manuscript.

## Declaration of Competing Interest

This work was supported by grants from the Ministry of Health, Labour and Welfare, Japan (19AA2007 and H30-Policy-Designated-004) and the Ministry of Education, Culture, Sports, Science and Technology, Japan (17H04141). The authors report no potential conflicts of interest.

## Appendix Supplementary materials

Image, application 1

Image, application 2
