# Supplementary material for: Intensive care unit versus high-dependency care unit for mechanically ventilated patients with pneumonia: a nationwide comparative effectiveness study
Source: Lancet Reg Health West Pac. 2021 Jul 5;13:100185. doi: 10.1016/j.lanwpc.2021.100185 (PMC8350066; doi:10.1016/j.lanwpc.2021.100185)
Supplement: Supplementary file 2 [file mmc2.docx]

**SUPPLEMENTARY MATERIAL**

**Intensive care unit versus high-dependency care unit for mechanically ventilated patients with pneumonia: a nationwide comparative effectiveness study**

Hiroyuki Ohbe, MD^1^, Yusuke Sasabuchi, PhD^2^, Hayato Yamana, PhD^1^, Hiroki Matsui, MPH^1^, Hideo Yasunaga, PhD^1^

^1^Department of Clinical Epidemiology and Health Economics, School of Public Health, The University of Tokyo, Tokyo, Japan

^2^Data Science Center, Jichi Medical University, 3311-1 Yakushiji, Shimotsuke, Tochigi Prefecture, 3290498, Japan

**List of Supplemental Tables and Figures**

**Supplemental Tables:**

**Supplemental Table 1** Japanese medical procedure codes used to define ICUs and HDUs

**Supplemental Table 2** The numbers of the patients in each pneumonia code

**Supplemental Table 3** Characteristics and outcomes for patients with differential distances of ≥ 0 km and < 0 km for the instrumental variable analysis in the main analysis

**Supplemental Figures:**

**Supplemental Figure 1** Distributions of propensity scores before propensity score matching in the main analysis

**Supplemental Figure 2** Distributions of propensity scores after propensity score matching in the main analysis

**Supplemental Figure 3** Balance of the covariates before and after propensity score matching in the main analysis

**Supplemental Figure 4** Balance of the covariates before and after overlap weighting in the main analysis

**Supplemental Table 1** Japanese medical procedure codes used to define ICUs and HCUs

|  |  |  | Cost per day, | Nurse-to-patient |
| --- | --- | --- | --- | --- |
| Name | Code | Description | USD* | ratio |
| ICU | A3002 | Emergency and critical care unit management fee 2 | 1072 | 1 to 2 |
| ICU | A3004 | Emergency and critical care unit management fee 4 | 1072 | 1 to 2 |
| ICU | A3011 | ICU management fee 1 | 1292 | 1 to 2 |
| ICU | A3012 | ICU management fee 2 | 1292 | 1 to 2 |
| ICU | A3013 | ICU management fee 3 | 882 | 1 to 2 |
| ICU | A3014 | ICU management fee 4 | 882 | 1 to 2 |
| HDU | A3001 | Emergency and critical care unit management fee 1 | 929 | 1 to 4 |
| HDU | A3003 | Emergency and critical care unit management fee 3 | 929 | 1 to 4 |
| HDU | A301-21 | HDU management fee 1 | 623 | 1 to 4 |
| HDU | A301-22 | HDU management fee 2 | 386 | 1 to 5 |

*Cost per day is shown for the first 7 days after ICU/HDU admission.

ICU, intensive care unit; HDU, high-dependency care unit; USD, United States dollars

**Supplemental Table 2** The numbers of the patients in each pneumonia code

|  |  | Number of |
| --- | --- | --- |
| ICD-10 codes | Description | patients |
| Typical bacteria |  |  |
| J13 | Pneumonia due to Streptococcus pneumoniae | 1,333 (9.0%) |
| J14 | Pneumonia due to Haemophilus influenzae | 231 (1.6%) |
| J150 | Pneumonia due to Klebsiella pneumoniae | 254 (1.7%) |
| J151 | Pneumonia due to Pseudomonas | 356 (2.4%) |
| J152 | Pneumonia due to staphylococcus | 744 (5.0%) |
| J153 | Pneumonia due to streptococcus, group B | 7 (0.1%) |
| J154 | Pneumonia due to other streptococci | 127 (0.9%) |
| J155 | Pneumonia due to Escherichia coli | 51 (0.3%) |
| J156 | Pneumonia due to other Gram-negative bacteria | 178 (1.2%) |
| J158 | Other bacterial pneumonia | 215 (1.5%) |
| J159 | Bacterial pneumonia, unspecified | 3,848 (25.9%) |
| J170 | Pneumonia in bacterial diseases classified elsewhere | 57 (0.4%) |
| Atypical bacteria |  |  |
| A481 | Legionnaires disease | 134 (0.9%) |
| J157 | Pneumonia due to Mycoplasma pneumoniae | 61 (0.4%) |
| J160 | Chlamydial pneumonia | 10 (0.1%) |
| Viruses |  |  |
| B012 | Varicella pneumonia | 0 (0.0%) |
| B052 | Measles complicated by pneumonia | 0 (0.0%) |
| J100 | Influenza with pneumonia, seasonal influenza virus identified | 12 (0.1%) |
| J110 | Influenza with pneumonia, virus not identified | 190 (1.3%) |
| J120 | Adenoviral pneumonia | 0 (0.0%) |
| J121 | Respiratory syncytial virus pneumonia | 3 (0.0%) |
| J122 | Parainfluenza virus pneumonia | 0 (0.0%) |
| J123 | Human metapneumovirus pneumonia | 1 (0.0%) |
| J128 | Other viral pneumonia | 1 (0.0%) |
| J129 | Viral pneumonia, unspecified | 9 (0.1%) |
| J171 | Pneumonia in viral diseases classified elsewhere | 6 (0.0%) |
| Fungus, parasite, and others |  |  |
| B371 | Pulmonary candidiasis | 18 (0.1%) |
| B59 | Pneumocystosis | 124 (0.8%) |
| J168 | Pneumonia due to other specified infectious organisms | 1 (0.0%) |
| J172 | Pneumonia in mycoses | 8 (0.1%) |
| J173 | Pneumonia in parasitic diseases | 91 (0.6%) |
| J178 | Pneumonia in other diseases classified elsewhere | 8 (0.1%) |
| Pathogen-nonconfirmed |  |  |
| J180 | Bronchopneumonia, unspecified | 914 (6.2%) |
| J181 | Lobar pneumonia, unspecified | 586 (3.9%) |
| J182 | Hypostatic pneumonia, unspecified | 12 (0.1%) |
| J188 | Other pneumonia, organism unspecified | 123 (0.8%) |
| J189 | Pneumonia, unspecified | 5,283 (35.6%) |

ICD-10, International Classification of Diseases, Tenth Revision

**Supplemental Table 3** Characteristics and outcomes for patients with differential distances of ≥ 0 km and < 0 km for the instrumental variable analysis in the main analysis

|  | Differential | Differential |  |
| --- | --- | --- | --- |
|  | distance ≥ 0 km | distance < 0 km |  |
| Characteristics and outcomes | (n = 7,103) | (n = 7,756) | ASD |
| ICU admission on the day of admission, n (%) |  |  | 74 |
| Age, years, mean (SD) | 75 (15) | 76 (14) | 9 |
| Male, n (%) | 4,687 (66) | 5,082 (66) | 1 |
| Smoking history, n (%) |  |  |  |
| Nonsmoker | 3,508 (49) | 3,832 (49) | 0 |
| Current/past smoker | 2,272 (32) | 2,484 (32) | 0 |
| Unknown | 1,323 (19) | 1,440 (19) | 0 |
| Body mass index at admission, kg/m^2^, n (%) |  |  |  |
| < 18.5 | 1,982 (28) | 2,078 (27) | 3 |
| 18.5–24.9 | 3,076 (43) | 3,343 (43) | 0 |
| 25.0–29.9 | 784 (11) | 869 (11) | 1 |
| ≥ 30.0 | 297 (4) | 313 (4) | 1 |
| Missing | 964 (14) | 1,153 (15) | 4 |
| Charlson comorbidity index, mean (SD) | 1.4 (1.4) | 1.4 (1.4) | 1 |
| Comorbidities, n (%) |  |  |  |
| Congestive heart failure | 2,260 (32) | 2,533 (33) | 2 |
| Malignancy | 490 (7) | 561 (7) | 1 |
| Metastatic solid tumor | 58 (1) | 60 (1) | 1 |
| Physical function at admission, n (%) |  |  |  |
| Total/severe dependence (Barthel Index 0–60) | 5,057 (71) | 5,906 (76) | 11 |
| Slight/moderate dependence (Barthel Index 61–99) | 127 (2) | 169 (2) | 3 |
| Independent (Barthel Index 100) | 548 (8) | 590 (8) | 0 |
| Missing | 1,371 (19) | 1,091 (14) | 14 |
| Cognitive function before admission, n (%) |  |  |  |
| No dementia | 4,983 (70) | 5,428 (70) | 0 |
| Mild dementia | 1,139 (16) | 1,271 (16) | 1 |
| Moderate/severe dementia | 981 (14) | 1,057 (14) | 1 |
| Home medical care before admission, n (%) | 790 (11) | 892 (12) | 1 |
| Ambulance use, n (%) | 5,800 (82) | 6,306 (81) | 1 |
| Admission on a weekend, n (%) | 1,903 (27) | 1,991 (26) | 3 |
| Location before hospitalization, n (%) |  |  |  |
| Home | 5,545 (78) | 6,033 (78) | 1 |
| Other hospitals | 891 (13) | 947 (12) | 1 |
| Nursing home | 667 (9) | 776 (10) | 2 |
| Cause of pneumonia, n (%) |  |  |  |
| Typical bacteria | 3,585 (50) | 3,727 (48) | 5 |
| Atypical bacteria | 102 (1) | 103 (1) | 1 |
| Viruses | 114 (2) | 107 (1) | 2 |
| Fungus, parasite, and others | 117 (2) | 130 (2) | 0 |
| Pathogen-nonconfirmed | 3,185 (45) | 3,689 (48) | 6 |
| Community-acquired pneumonia, n (%) | 6,379 (90) | 7,019 (90) | 0 |
| Severity of pneumonia, n (%) |  |  |  |
| Blood urea nitrogen >21 mg/dl or dehydration | 4,757 (67) | 5,014 (65) | 5 |
| Oxygenation |  |  |  |
| SpO_2_ > 90% in room air | 1,045 (15) | 1,080 (14) | 2 |
| SpO_2_ > 90% in FiO_2_ < 35% | 1,637 (23) | 1,882 (24) | 3 |
| SpO_2_ > 90% in FiO_2_ ≥ 35% | 4,421 (62) | 4,794 (62) | 1 |
| Impaired consciousness | 4,029 (57) | 4,270 (55) | 3 |
| Systolic blood pressure < 90 mmHg | 2,045 (29) | 2,116 (27) | 3 |
| A-DROP score, mean (SD) | 1,276 (18) | 1,301 (17) | 3 |
| Immunocompromised, n (%) | 3,426 (48) | 3,479 (45) | 7 |
| CRP ≥ 20mg/dl or lung infiltration, n (%) | 3.1 (1.3) | 3.0 (1.2) | 2 |
| Organ support therapies on the day of admission, n (%) | |  |  |
| Dopamine | 806 (11) | 724 (9) | 7 |
| Dobutamine | 347 (5) | 272 (4) | 7 |
| Noradrenaline | 1,855 (26) | 1,615 (21) | 13 |
| Adrenaline | 691 (10) | 793 (10) | 2 |
| Vasopressin | 227 (3) | 182 (2) | 5 |
| Cardiac massage | 497 (7) | 703 (9) | 8 |
| Defibrillation | 86 (1) | 96 (1) | 0 |
| Red blood cell transfusion | 283 (4) | 247 (3) | 4 |
| Fresh frozen plasma transfusion | 96 (1) | 81 (1) | 3 |
| Platelet transfusion | 48 (1) | 29 (0) | 4 |
| Albumin | 393 (6) | 275 (4) | 10 |
| Renal replacement therapy | 268 (4) | 242 (3) | 4 |
| Extracorporeal membrane oxygenation | 34 (0) | 21 (0) | 3 |
| Hospital characteristics, n (%) |  |  |  |
| Teaching hospital | 6,608 (93) | 7,193 (93) | 1 |
| Tertiary emergency hospital | 4,011 (56) | 5,268 (68) | 24 |
| Academic hospital | 1,278 (18) | 881 (11) | 19 |
| Total number of hospital beds |  |  |  |
| Low (24–381 beds) | 1,948 (27) | 1,827 (24) | 9 |
| Medium low (382–522 beds) | 1,713 (24) | 1,956 (25) | 3 |
| Medium high (523–680 beds) | 1,675 (24) | 2,029 (26) | 6 |
| High (681–1,334 beds) | 1,767 (25) | 1,944 (25) | 0 |
| Outcomes |  |  |  |
| 30-day in-hospital mortality, n (%) | 1,860 (26.2) | 2,351 (30.3) |  |
| ICU/HDU mortality, n (%) | 1,189 (16.7) | 1,551 (20.0) |  |
| Length of hospital stay, days, mean (SD) | 29 (34) | 28 (39) |  |
| ICU/HDU-free days, days, mean (SD) | 17 (11) | 16 (11) |  |
| MV-free days, days, mean (SD) | 15 (12) | 14 (12) |  |
| Total hospitalization cost, USD, mean (SD) | 17,347 (15,746) | 15,872 (16,080) |  |
| ARDS after admission, n (%) | 83 (1.2) | 82 (1.1) |  |
| CRBSI after admission, n (%) | 9 (0.1) | 18 (0.2) |  |

ICU, intensive care unit; HDU, high-dependency care unit; ASD, absolute standardized mean difference; SD, standard deviation; A-DROP, age, dehydration, respiration, disorientation, and blood pressure; CRP, C-reactive protein; MV, mechanical ventilation; USD, United States dollars; ARDS, acute respiratory distress syndrome; CRBSI, catheter related blood stream infection

**Supplemental Figure 1** Distributions of propensity scores before propensity score matching in the main analysis


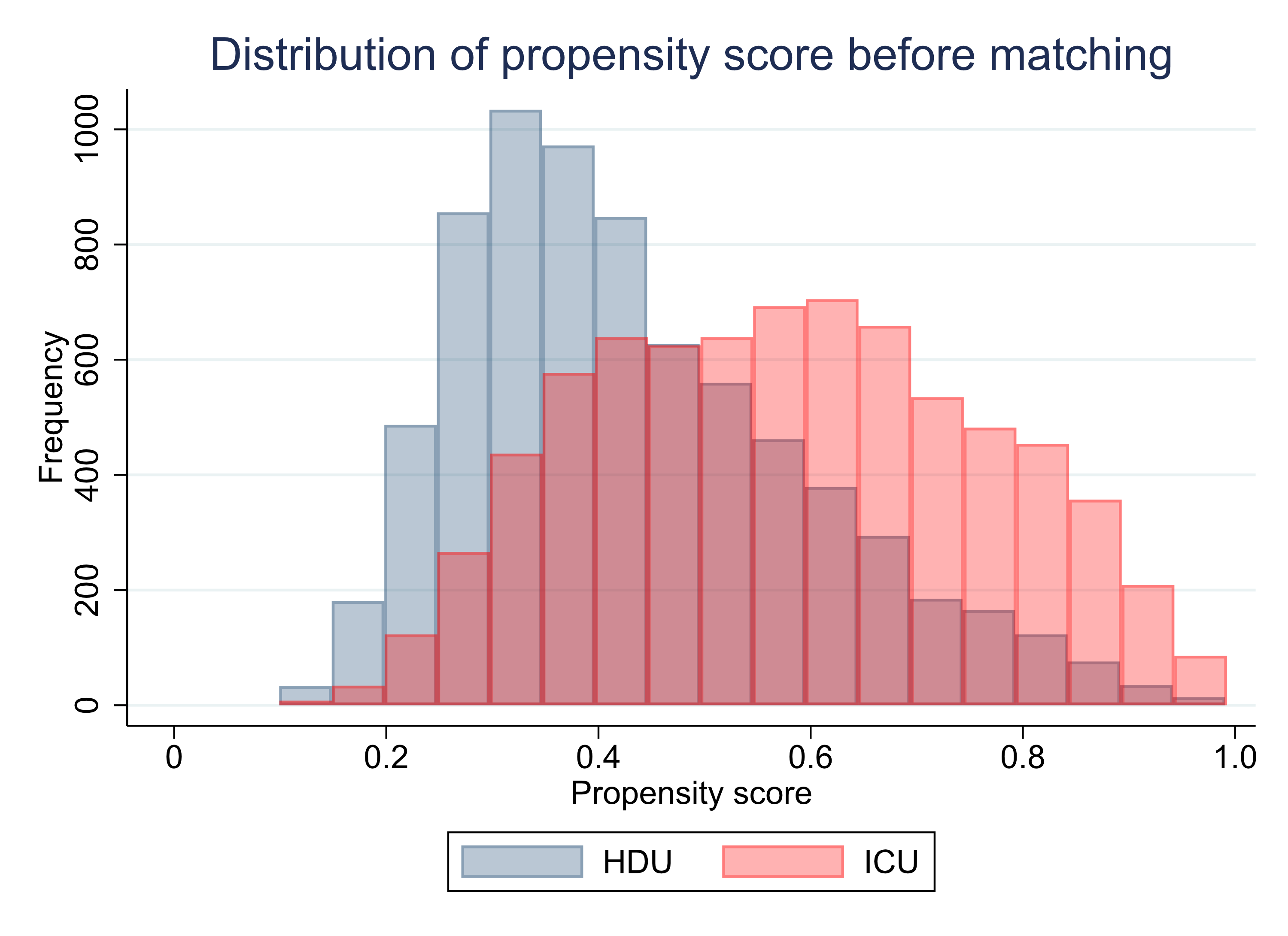


ICU, intensive care unit; HDU, high-dependency care unit

**Supplemental Figure 2** Distributions of propensity scores after propensity score matching in the main analysis


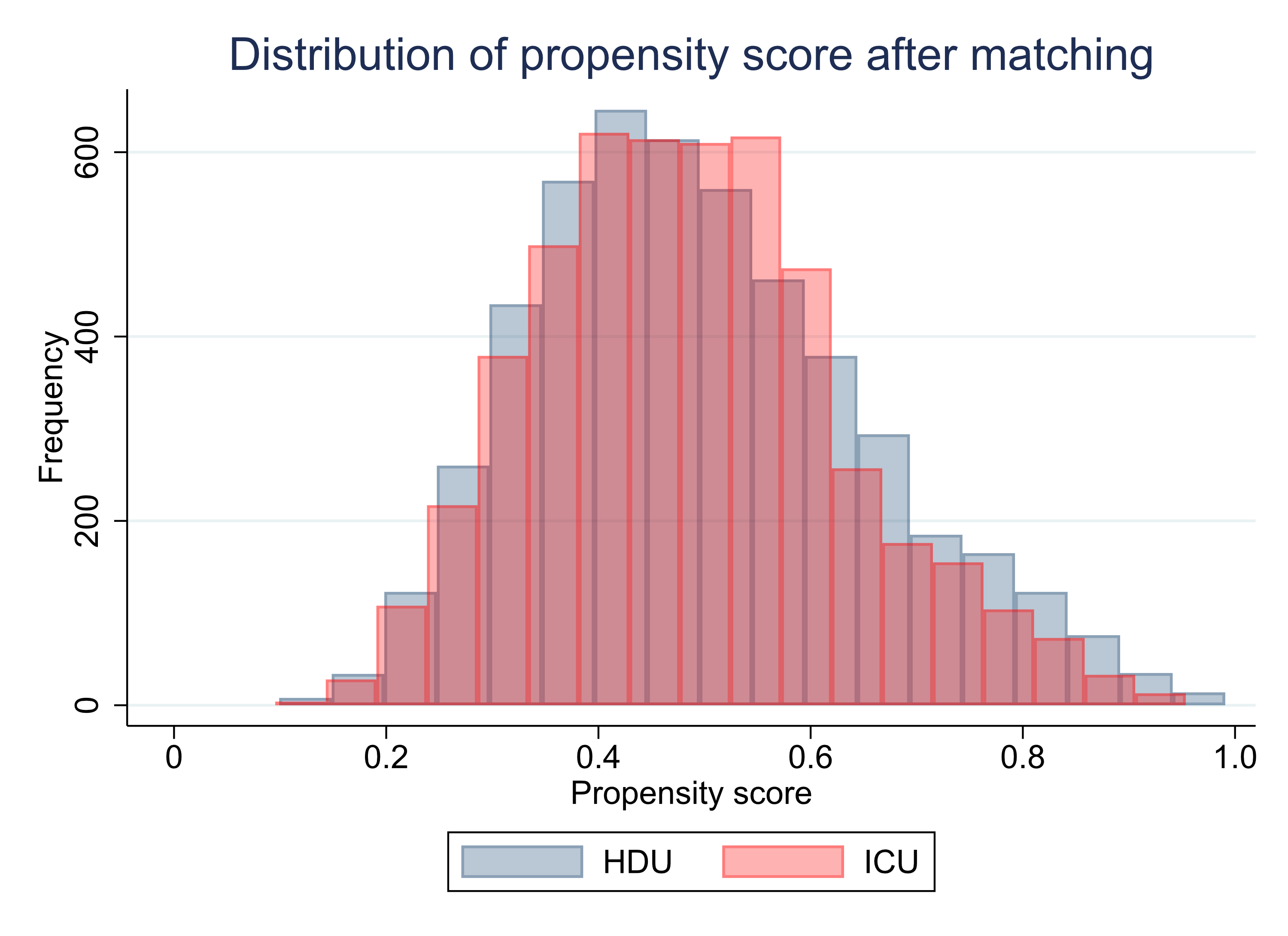


ICU, intensive care unit; HDU, high-dependency care unit

**Supplemental Figure 3** Balance of the covariates before and after propensity score matching in the main analysis

A-DROP, age, dehydration, respiration, disorientation, and blood pressure; CRP, C-reactive protein

**Supplemental Figure 4** Balance of the covariates before and after overlap weighting in the main analysis

A-DROP, age, dehydration, respiration, disorientation, and blood pressure; CRP, C-reactive protein
